# Functional and Phylogenetic Implications of Canopy Mesophication in Temperate Hardwood Forests

**DOI:** 10.1002/ece3.73323

**Published:** 2026-04-02

**Authors:** Sam W. Anderson, David A. Rogers, Katherine A. McCulloh

**Affiliations:** ^1^ Madison Botany Department University of Wisconsin Madison Wisconsin USA; ^2^ Parkside Biology Department University of Wisconsin Kenosha Wisconsin USA

**Keywords:** community composition, functional shifts, mixed‐modeling, phylogenetic clustering

## Abstract

Forest mesophication describes the shifting of plant communities from fire‐adapted, drought tolerant systems to those dominated by species better adapted to shaded, closed‐canopy environments. While commonly characterized from a taxonomic perspective, the phylogenetic underpinnings and functional attributes of forest mesophication have received less quantitative testing. To address this, we applied phylogenetic and functional trait data to legacy datasets of changing forest composition in Wisconsin, USA from the latter half of the 20th century. In both the 1950s and 2000s, forests demonstrated a phylogenetically clustered structure, with closely related species occupying similar sites. The inclusion of functional traits into mixed models accounted for 40.8% of the variance explained by this clustering in the 1950s and just 19.6% in the 2000s. This diminished explanatory value of functional traits corresponds with significant functional and phylogenetic turnover, with the majority of sites demonstrating substantial temporal functional beta diversity. Contemporary trait syndromes were defined by broad declines in drought tolerance, fire tolerance, and litter flammability, as well as an increase in leaf traits associated with acquisitive resource strategies. Such shifts identify the diminished capacity of traits to explain phylogenetic clustering, but species‐level trends and landscape‐level functional trajectories also give stewards explicit targets to meet land management goals. While future studies on the influence of intraspecific trait variation and trait‐environment relationships would further clarify functional shifts in temperate deciduous forests, our findings provide quantifiable support for the hypothesized functional implications of mesophication.

## Graphical abstract

Mesophication describes the shifting of forests fire‐adapted systems to those dominated shaded‐adapted species, but the functional underpinnings of forest mesophication have received less quantitative testing. Using 20th century legacy datasets of changing forest composition in Wisconsin, USA, we characterized broad declines in drought tolerance, fire tolerance, and litter flammability, as well as an increase in leaf traits associated with acquisitive resource strategies. This quantifies species‐level trends and landscape‐level functional trajectories that give stewards explicit targets to meet land management goals.

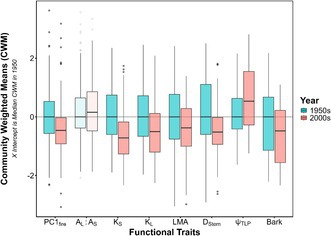

## 1 Introduction

Spanning much of the northern hemisphere, temperate deciduous forests are one of the largest forest biomes globally (Adams et al. 2019; Dreiss and Volin 2020). Trees within temperate deciduous forests are well adapted to varying annual precipitation and intense seasonality, but they are also increasingly exposed to elevated temperatures and altered precipitation regimes globally (Allen et al. 2015; Bréda et al. 2006; Hoffmann et al. 2011; Marchin et al. 2010). Temperate forests have noticeably changed in terms of community composition, with many forests shifting toward a species composition adapted to hotter, drier environments (Allen et al. 2010; Delzon et al. 2013; Fernández‐Manjarrés et al. 2018). However, this trend in aridification is not ubiquitous. One hypothesized compositional trend for temperate forests in North America is mesophication, with forests moving away from fire‐prone, fire‐adapted communities toward a more shaded, fire‐intolerant community (Alexander et al. 2021; Hanberry 2019; Nowacki and Abrams 2008). Understanding how temperate forests are changing in response to global, regional, and local factors improves our understanding of forest management and climate adaptation (Swanston et al. 2018), but addressing such questions requires robust data on both community composition and the life histories of temperate trees (Rogers et al. 2017, 2008).

The temperate forests of eastern North America, specifically the well‐surveyed forests of the prairie‐forest ecotone in Wisconsin (WI), are uniquely well‐placed for assessing the proposed ecological trajectory of mesophication (Rogers et al. 2008). Descriptions of the pre‐colonization ecology, surveys of hundreds of forests in the 1950s (Curtis 1959), and resurveys of those same forests in the 2000s serve as a robust baseline for documenting shifts in temperate forest composition (Paciorek et al. 2021; Shea et al. 2014; Waller et al. 2013). In WI temperate forests, the drivers of mesophication are primarily hypothesized to be both fire suppression and climatic shifts in temperature and precipitation (Nowacki and Abrams 2008). Regular, low‐intensity fires, both natural and anthropogenic, were historically common throughout the prairie‐forest ecotone, integral in supporting understory biodiversity and overstory structure and composition (Alstad and Damschen 2016; Curtis 1959; Leach and Givnish 1999; Reich et al. 1990a; Wolf 2004). While fire consistently shaped the natural communities of the region for millennia after glaciation, the use of prescribed fire to manage these systems almost completely fell out of practice in the early twentieth century (Curtis 1959; Wolf 2004). Along with shifts in fire regime, climatic shifts in the twentieth century have altered conditions for temperate deciduous forests. For the eastern US, both twentieth‐century climatic trends and climate predictions highlight increasing annual precipitation and increasing temperatures (Hayhoe et al. 2008; Kucharik et al. 2010; Kucharik and Serbin 2008), with Wisconsin annual precipitation increasing 13.97 mm/decade on average, and mean annual temperature increasing 0.11°C/decade (National Centers for Environmental Information).

This climatic and ecological context for mesophication has motivated the research of temperate broadleaf forests for much of the last two decades and is well supported by empirical data on compositional changes (Alexander et al. 2021; Nowacki and Abrams 2008). Often, ecological characterization of individual tree species as ‘mesophytes’ (e.g., *Acer saccharum* Marshall) and ‘pyrophytes’ (e.g., *Quercus macrocarpa* Michx.) has been in terms of their realized ecological niche, specifically in terms of their relative abundance and dominance compared to soil fertility (Alexander et al. 2021; Babl et al. 2020; Curtis 1959; H. Gleason 1926; Hanberry 2019). More recent work has honed our mechanistic understanding of mesophication in the form of trait‐based studies of trees characterized as mesophytes or pyrophytes, significantly improving our ability to generalize proposed trends in temperate forest turnover more broadly (Iverson et al. 2019; Swanston et al. 2018). Such analyses are particularly pertinent now that the collection of environmental and ecological data can be coupled with more readily available functional trait data (Amatangelo et al. 2014; Beck et al. 2022; Li and Waller 2017).

In characterizing changes in temperate forests, integrating plant functional traits into abundance‐based analyses provides a more mechanistic understanding of species' relationships with environmental parameters and directly tests the importance of such traits on abundance metrics (Bolker et al. 2009; ter Braak 2019). When viewed over time, functional shifts (i.e., functional beta diversity) can corroborate or refute proposed mechanisms through which forests persist or change when facing altered disturbance regimes, competition dynamics, or climatic shifts (Aubin et al. 2016, 2018; Legendre 2019). Canopy composition both responds to (i.e., response traits) and drives changes (i.e., effect traits) in environmental conditions, altering light availability, soil moisture, nutrient cycling, and fine fuel accumulation, with many functional traits being a crucial factor in determining how species differ in shaping understory environment (Bennett and Klironomos 2019; Scavotto et al. 2024; Varner et al. 2021). If WI forests are indeed becoming more mesic, then functional signatures should be present within legacy datasets that document shifting community composition. Community level signatures would be detectable in shifts toward thinner bark, species with less negative leaf turgor loss point (Ψ_TLP_), foliar traits associated with more acquisitive resource strategies like low leaf mass per area (LMA), litter traits with reduced flammability, more efficient transport of water, and lower wood density (D_stem_) (Alexander et al. 2021; Díaz et al. 2016). While there are clear shifts in WI canopy communities in terms of composition, the shifts in functional trait representation and the extent to which individual species drive such shifts remain unquantified (D. A. Rogers et al. 2008).

While traits provide insight into species' survival strategies, trait‐based analyses often fail to capture all of the important facets related to a species' ability to grow, persist, and reproduce in a given environment (Li and Ives 2017). Phylogenetic beta diversity can be used to detect shifts in composition that may be unquantified by functional trait data. By including phylogenetic covariance in a mixed modeling approach, we can also characterize which traits display phylogenetic signal (L. D. Anderegg et al. 2018), account for any generated covariance from unmeasured traits with phylogenetic signal (Ives and Helmus 2011; Revell 2010), improve coefficient estimates, and account for variation in the residuals of mixed models that incorporate traits (Li and Ives 2017).

Including phylogenetic co‐variance in mixed modeling also allows for the testing of various hypotheses related to the selective pressures of mesophication on phylogenetic clustering (i.e., closely related species tending to co‐occur). Specifically, (i) forests could be moving away from a phylogenetically clustered community structure. Historic fire regimes could have limited the regional species pool to a group of fire‐adapted species, such as oaks or various resprouting shrubs, with fire‐intolerant species only clustered on sites that rarely burned. With fire suppression, more phylogenetically distant species would have moved into these oak‐dominated forests, reducing the amount of variation explained by phylogenetic clustering in later resurveys. (ii) An increasing abundance of ‘mesophytes’ could create more shaded forest environments that favor closely related species adapted to low light levels, such as members of genus *Acer* or family *Betulaceae* common in forest understories. In this scenario, an increasing amount of phylogenetic clustering would be seen as most forests progress toward communities consisting mostly of mesophytes, leaving more ‘pyrophytic’ dry forest species on only the most thin‐soiled, exposed locations. (iii) Both surveys could demonstrate phylogenetic clustering, with the significance of trait fixed effects on frequency from mixed modeling revealing whether 2000s clustering is novel or reflects the clustering seen in the 1950s. Given the availability of ultrametric phylogenies for flowering plants in Wisconsin (Spalink et al. 2018), we can readily test these hypothesized phylogenetic patterns associated with the process of mesophication. Such models can also directly test the effect of functional traits on species frequency across time and within any phylogenetic clustering identified (Givnish et al. 2020). However, clarifying assumptions for how traits vary within and among species is key to assessing their adaptive significance before testing for contribution to forest mesophication (Di Biase and Fattorini 2025; Westerband et al. 2021).

Considering these perspectives, the driving motivation of this analysis is to (i) discern the functional trajectories and phylogenetic clustering of overstories in southern Wisconsin forests and (ii) determine whether the functional shifts observed support hypothesized patterns of forest mesophication. To do so, we combine robust forest survey and resurvey data, a recently created regional phylogeny of flowering plants, and a functional trait dataset of dominant native tree species present in WI forests. We hypothesize that forest overstories are functionally and phylogenetically dissimilar from those measured in the 1950s, driven primarily by increases in the frequency of *Acer* individuals and declines in *Quercus* individuals. Given large‐scale alterations to historical fire regimes that had prevented more mesic species from establishing in many forests, we predict that there will be reduced phylogenetic clustering between sites sampled in the 2000s when compared to sites sampled in the 1950s, providing a rational basis for shifts in functional strategies in WI forests. Likewise, we predict that plant traits related to fire tolerance (bark thickness), drought tolerance (Ψ_TLP_), and an acquisitive growth strategy (LMA, stem hydraulics, D_stem_) will account for much of the variance explained by patterns of phylogenetic clustering (Table 1). At the community level, we predict that community‐weighted trait values will shift toward a less fire‐adapted, less drought‐tolerant, more acquisitive functional strategy used to describe ‘mesic’ species (Table 1). By combining community, phylogenetic, and trait data, our goal is to provide sound functional and phylogenetic evidence in support of forest mesophication at the regional and decadal scale, while simultaneously identifying species‐specific impacts on forest communities to inform temperate deciduous forest management and conservation.

**TABLE 1:** A summary of the various analyses performed on community composition data.

Scale at which the factor of interest is applied	Number of replicates at the appropriate scale	Analysis	Citation
Sites/Site‐pairs	138 sites/Site‐pairs	TBI, Pairwise Functional Similarity, CWM Analysis (1000× permutations)	(Legendre 2019), (Li 2018), (Li and Waller 2017)
Species	19 Species	Generalized linear mixed models (GLMM)	(K. R. Clarke 1993; M. M. Jackson et al. 2012)
Site*Species	2662 Site*Species combinations	Phylogenetic generalized linear mixed models (PGLMM)	(Li et al. 2017; Li and Ives 2017)

*Note:* Factors of Interest: Site‐level shifts in diversity/community‐weighted mean (CWM) values, species representation over time, and species‐site frequency as modeled by PGLMM.

## 2 Materials and Methods

### 2.1 Community Composition Surveys

The survey data used here were described in Curtis (1959) and Rogers et al. (2008). Briefly, sites in forests > 6.0 ha were originally selected in the 1950s based on minimal disturbance from logging, development, or agriculture, and met a Chi‐square test of homogeneity (Curtis 1959). At forty points in each site, two trees were used to characterize stand structure using the random pairs method (Curtis and McIntosh 1951). These points were generally 15–20 m apart on transects 600–1000 m in length, arranged in a square or ‘U’ shape (Curtis 1959; Waller et al. 2013). Resurveying efforts occurred at all sites with surviving tree canopies, including forests in partly residential areas that were at least 0.6 ha, using the same protocols as the 1950s (D. A. Rogers et al. 2008) (S1). Because sample sizes were larger in the 2000s than in 1950, a random subset of plots was chosen to match the original size and spatial structure of the 1950s surveys for 138 sites, standardized to 80 individuals per site (D. A. Rogers et al. 2008; Waller et al. 2013).

### 2.2 Trait Data

Trait data were compiled to produce species mean trait values for nineteen of the most common native tree species in the southern upland forest dataset. Native species were selected based on their quantified importance in southern forest composition (Bray and Curtis 1957; Peet and Loucks 1977). Species such as *Juglans cinerea* L. and *Fraxinus americana* L. were excluded from analyses due to increasing rarity in natural communities. Traits were included for analysis based on their importance in resource acquisition strategies of broadleaf deciduous species (Wright et al. 2004), ability to quantify woody plant hydraulics in the context of supporting these leaf acquisitive trait strategies (W. R. Anderegg et al. 2016; Reich 2014), the ability to characterize species' response to drought (Bartlett et al. 2014), and use in describing litter flammability (Varner et al. 2021). For analyses, we transformed skewed data in accordance with Box‐Cox transformation guidelines to meet assumptions of normality (Box and Cox 1964). Highly skewed leaf area (LA) data were log‐transformed, while leaf area: sapwood area ratios (A_L_A_S_) and leaf area‐specific maximum stem hydraulic conductivity (K_L_) were square‐root transformed. All trait data were then Z‐transformed to have mean of zero and standard deviation of one, allowing for comparable interpretations of modeling coefficients. Additionally, the adaptive significance of trait values assumes significant trait variation among species (Mudrák et al. 2019; Shipley et al. 2016; Westerband et al. 2021). Using a mixed‐modeling approach with R package *lme4* (1.1–35.5), we partitioned the variance of the trait values assessed to justify the use of species‐mean trait values in our analyses (Bates et al. 2014).

In August 2023, sun‐exposed branches ~1 cm in diameter were collected from eight individuals of each species from the naturally occurring temperature deciduous forests stewarded by the University of Wisconsin Arboretum, The Lakeshore Nature Preserve, or Hemlock Draw State Natural Area in southern Wisconsin. Samples were transported in humid plastic bags to the lab, where leaves were removed and placed in a damp bag and refrigerated, while stems were recut underwater, bagged in deionized water, and refrigerated. Five leaves per individual were used to calculate leaf area (LA) and leaf mass per area (LMA) per standardized methods (Pérez‐Harguindeguy et al. 2013). Bulk leaf samples were placed in a 65°C oven to dry for 48 h to calculate dry mass.

Turgor loss point (Ψ_TLP_) was quantified using vapor pressure osmometry, allowing for the rapid quantification of osmolality (mmol/kg) and subsequent calculations of Ψ_TLP_ (Bartlett et al. 2012). Leaf discs from hydrated leaves were frozen in liquid nitrogen (2 min), punctured ten times with sharp forceps, and placed in the Vapro 5600 vapor pressure osmometer (ELI Tech Group Inc.; Logan, Utah). After equilibrating (10 min) in the chamber, osmolality was measured and converted using the equations provided by Bartlett et al. (2012). To measure branch hydraulics, stem segments were submerged in 20 mM KCl solution and placed under a dynamic vacuum for 30 min until bubbles were no longer visible, removing any embolisms. The open‐source software *conductR* in RStudio (DD Smith 2018) was used to record the flow rate of KCl solution through a stem segment of known length under conditions of no pressure head and a pressure head of known height to calculate maximum hydraulic conductivity (K, g·mm·s^−1^·MPa^−1^) (Sperry et al. 1988). Stem and pith diameter were converted to areas assuming circularity and then used to adjust K to xylem cross‐sectional area‐specific hydraulic conductivity (K_S_, g·s^−1^MPa^−1^·mm^−1^). The bulk dry mass of all leaves attached to each branch was converted to bulk fresh area using LMA to calculate A_L_A_S_. K_S_ and A_L_A_S_ were then used to calculate leaf area‐specific maximum stem hydraulic conductivity (K_L_, g·s^−1^·MPa^−1^·m^−1^) (Sperry et al. 1988), which is valuable in understanding the maximum stem hydraulic supply for a given leaf area. Stem density (D_stem_) and sapwood density (D_sap_) were calculated from ~2 cm segments of stem tissue with bark removed. Sapwood density samples were split longitudinally, and pith tissue was removed with forceps. Volume measurements were obtained using volume displacement, and dry mass was obtained after drying for 48 h at 60°C. (Pérez‐Harguindeguy et al. 2013).

Senesced leaf tissue for each tree species was collected in the fall of 2023 to perform flammability analysis using methods outlined by Varner et al. (2021). Litter was dried at 60°C for 48 h, with at least three 15 g dry litter samples analyzed per species. From burn trials, maximum flame height (cm), flame duration (s), smolder duration (s), percent consumed (%), consumption rate (g*s^−1^), and fireline intensity (kW·m^−1^) can be derived (Varner et al. 2021). Skewed data were log‐transformed prior to principal components analysis. The first principal component (PC1_Fire_) score was extracted for each species, with a larger PC1_fire_ score corresponding to more intense, rapid, and consumptive fire behavior (S2). Bark thickness data collected from 21 to 27 individuals per species were obtained from collaborators and used to model bark thickness per species for a 10 cm diameter at breast height individual (Ava Copple and Steven Augustine, unpublished data).

### 2.3 Statistical Methods

While alpha diversity can characterize dynamics within communities, using beta diversity to describe the differences in community composition between survey efforts can inform our understanding of plant community turnover temporally. Using Bray–Curtis dissimilarity, a Temporal Beta Diversity Index (TBI), ranging from 0 (no change) to 1 (completely novel community) provides a quantitative basis for how dissimilar communities are compared to original surveying (Legendre 2019). Such differences can be further partitioned into changes driven by gaining new species (TBIgain) or losing originally present species (TBIloss) using the R package *adespatial* (version 0.3–24). To corroborate TBI, intercept‐only generalized linear mixed models (glmm) using a zero‐inflated Poisson distribution were used to determine species‐level intercepts for frequency in both 1950s and 2000s (Beck et al. 2022). These intercepts reflect the ‘average’ frequency of a species across all sites, with smaller numbers denoting more infrequent species, and comparisons of coefficients between 1950s and 2000s highlighting species that are increasing or decreasing in frequency over time. To assess functional and phylogenetic beta diversity, we utilized the R package *hillR* (version 0.5.2). Using methods accounting for varying frequency of species, we calculated the functional and phylogenetic similarity and standardized functional beta diversity to between 0 (completely dissimilar) and 1 (completely similar) at each site, using *hill_func_parti* and *hill‐phylo‐parti* with a Hill's number (*q* = 1) that accounts for varying species' frequency (Chao et al. 2014; Chiu and Chao 2014). Since taxonomic diversity can be highly correlated with functional or phylogenetic diversity, we compared functional and phylogenetic similarity of each site to null models by calculating standardized effect sizes (SES) (Botta‐Dukát 2018; Gotelli and McCabe 2002). For each site, trait data were randomized (999 permutations) among species while holding species' frequency constant within sites to generate a null distribution to compare with observed taxonomic and phylogenetic similarity.

### 2.4 Community Weighted Metrics

Assessing shifts in trait representation between the 1950s and 2000s was done using a community weighted means (CWM) framework. While the use of CWM to establish trait‐environment relationships has received recent attention for a propensity toward elevated Type 1 error, shifts in CWM that make no assumptions about how such traits may be driving changes in community composition still allow for the testing of functional shifts (Lepš and de Bello 2023; Miller et al. 2019). Previous analyses using CWM with similar legacy datasets permuted trait values 1000 times between the two survey datasets to derive statistical support for community shifts (Li and Waller 2017). We used a similar approach to test temporal shifts in forest traits related to aspects of stress tolerance and resource acquisition, in addition to a straightforward comparison between surveys using a Wilcoxon Sign‐rank test.

### 2.5 Mixed Modeling

Phylogenetic generalized linear mixed models (pglmm) from R package *phyr* (version 1.1.0) were used to model the distribution of individual species (Jackson et al. 2012), model the effects of species‐mean traits on species' frequency between survey efforts, and account for phylogenetic nonindependence in trait‐based analyses (Ives and Helmus 2011; Li et al. 2017; Gallinat and Pearse 2021). To test for the likelihood of closely related species occupying similar sites (phylogenetic clustering), a nested phylogenetic parameter was included in mixed models (Li et al. 2017). The contributions of various random effects were evaluated using a likelihood ratio test against a reduced model, while the fixed effects were assessed using the confidence interval of the posterior distributions (Li et al. 2020). Frequency models were fit using a Bayesian zero‐inflated poisson distribution, common for sparse datasets (Bolker et al. 2009; Brooks et al. 2017). Non‐zero frequency models were as follows:

$$Y_{i}=\beta_{0}+a_{\text{site}\left[i\right]}+c_{\mathrm{spp}\left[i\right]}+d_{\mathrm{spp}.\mathrm{phy}\left[i\right]}+\left(\beta_{1}+f_{\text{site}\left[i\right]}\right){\text{trait}}_{\mathrm{spp}\left[i\right]}+z_{i}$$

$a\sim \text{Gaussian}\;\left(0,\sigma_{\text{site}}^{2}I_{m}\right)–$Site level random effect allowing $\beta_{0}$ to vary between *m* sites (*1|site* in *phyr* syntax). $c\sim \text{Gaussian}\;\left(0,\sigma_{\mathrm{spp}}^{2}I_{n}\right)$–Species‐level random intercept that adjusts $\beta_{0}$ (global ‘average’ frequency for all species) between *n* species (*1|species*). $d\sim \text{Gaussian}\;\left(0,\sigma_{\mathrm{spp}}^{2}\Sigma_{\mathrm{phy}}\right)$–Species‐level random intercept that adjusts $\beta_{0}$ (global ‘average’ frequency) while accounting for species' phylogenetic relationships (*1|species__*). $f\sim \text{Gaussian}\;\left(0,\sigma_{\text{site}}^{2}I_{m}\right)$–A site level random effect that allows the slope of the relationship between frequency and *trait* to vary at each site (*trait|site*). $z\sim \text{Gaussian}\;\left(0,\text{kron}\left({I_{m},\sigma }_{z}^{2}\Sigma_{\mathrm{phy}}\right)\right)$–nested phylogenetic variance–covariance matrix within sites (*1|species__@site*). *β*_*0*_ specifies the average frequency across all species (global intercept). *β*_*1*_ allows the frequency to vary with the value of the trait. *Separate *β* terms can be constructed for multiple traits in the same model*.

We tested hypotheses of the presence of phylogenetic attraction in both 1950s and 2000s using the above equation without adding any functional traits. If significant phylogenetic attraction was observed, we then added functional traits to test whether they could account for the observed phylogenetic attraction patterns in the data. A forward model selection process was used in adding traits as fixed effects (Jamil et al. 2012; Li et al. 2017). Briefly, traits were included based on if they demonstrate phylogenetic conservatism via Pagel's *λ* (R package *phytools* version 2.3‐0) and improving of WAIC over null models that excluded phylogenetic random effects (Table S1). This resulted in models that included Ψ_TLP_, bark thickness, D_Stem_, LMA, and PC1_fire_ (Li et al. 2017).

## 3 Results

### 3.1 Community Compositional Shifts

**FIGURE 1:**
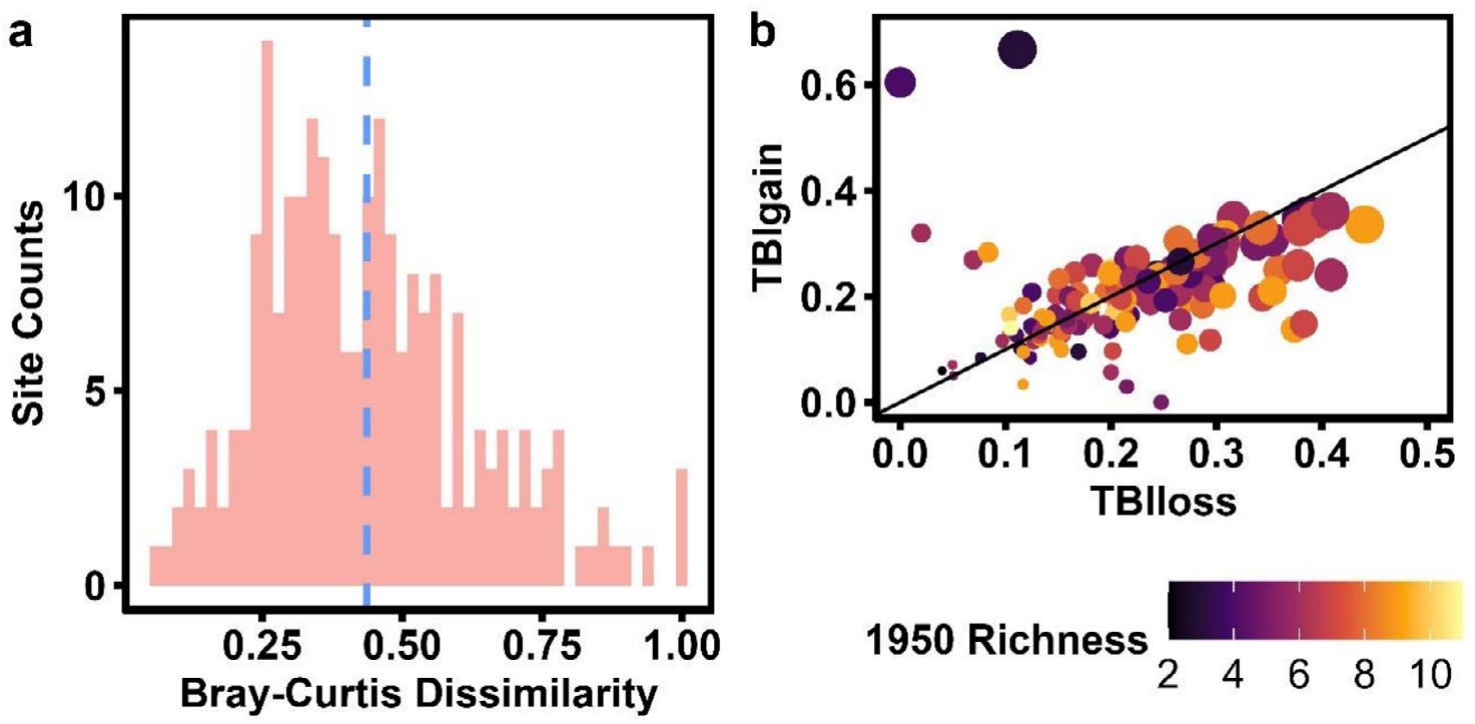
(a) A histogram of 138 site TBI dissimilarities between the 1950s survey effort and resurveys of the 2000s for southern Wisconsin forests, with a mean dissimilarity of 0.42. (b) Decomposition of TBI into components driven by species gains (TBIgain) and losses (TBIloss). There is no significant difference in mean TBIgain and TBIloss values for sites. Site color is denoted by the species richness of the canopy at each site when originally surveyed (1950s), while size denotes overall TBI value, with larger points having higher dissimilarity.

**FIGURE 2:**
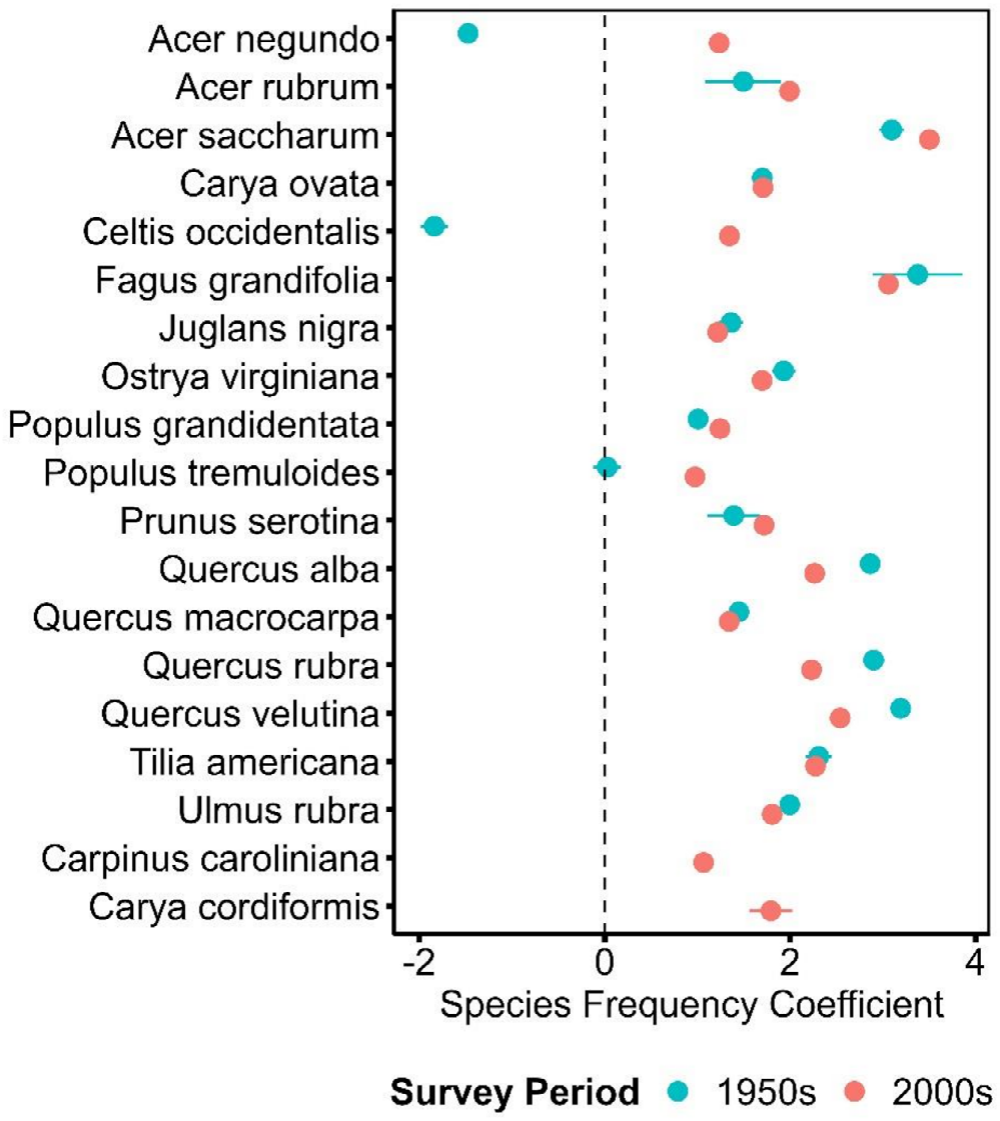
Species modeled coefficients for frequency in intercept‐only models using species and site as random effects. Lower coefficient values denote species that are less frequent across all sites. 2000s frequency coefficients for *Carpinus caroliniana* Walter and *Carya cordiformis* (Wangenh.) K.Koch are at the bottom of the figure due to the lack of representation in 1950s canopies.

**FIGURE 3:**
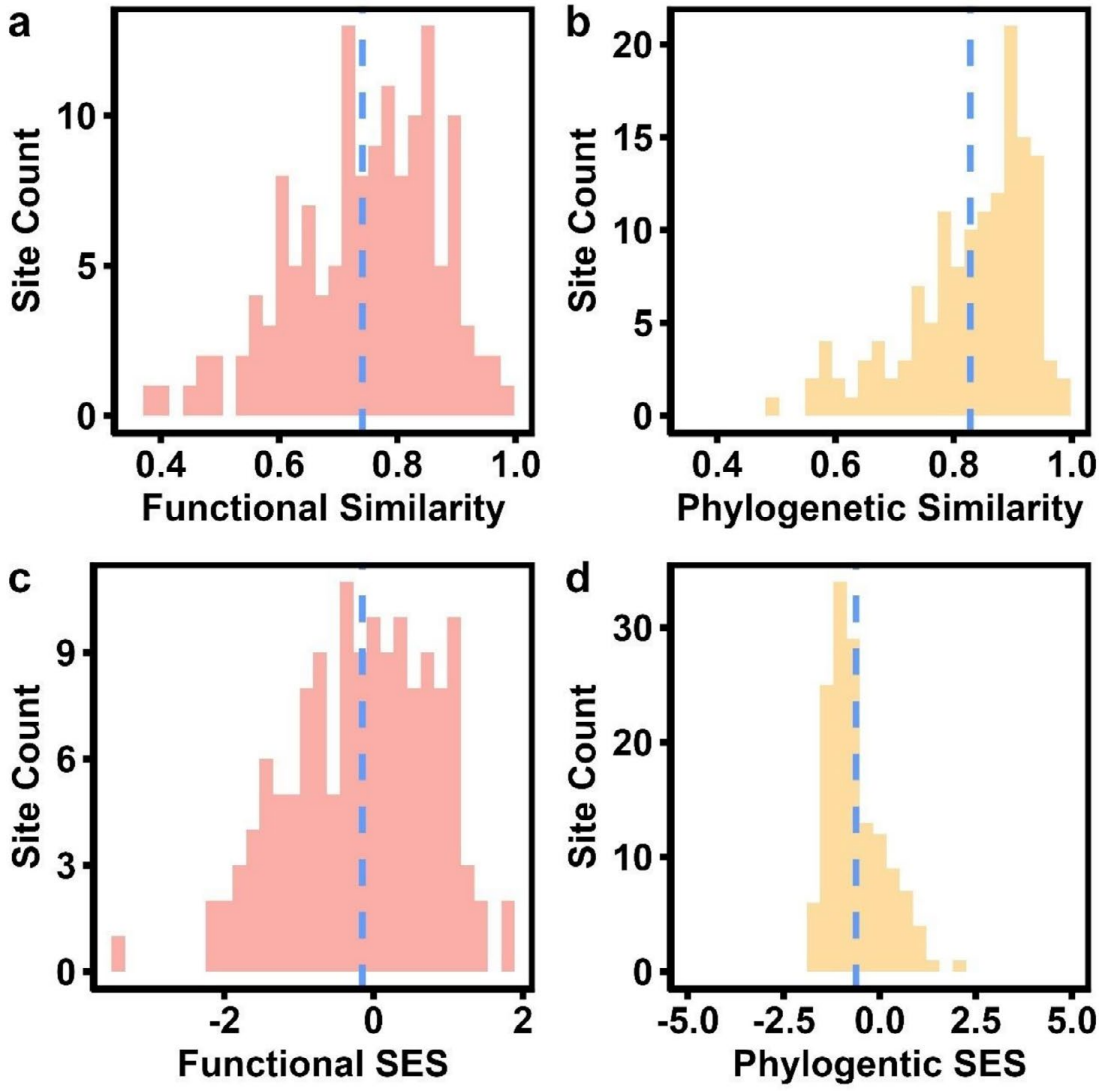
Histograms demonstrating functional (a) and phylogenetic similarity (b) scaled to between 0 and 1, with 1 being functionally/phylogenetically identical. Significantly negative Standardized Effect Sizes (SES) for both similarity metrics (c, d) were determined using a paired *t*‐test statistic.

Across all sites, taxonomic composition shifts significantly between the 1950s and 2000s, with an average pairwise site Bray–Curtis dissimilarity of 0.42, despite marginal shifts in species richness and Shannon diversity (Figures 1a, S3). Though moderate dissimilarity is seen in southern WI forests, there is no significant difference in the proportion of dissimilarity driven by species losses or species gains overall (*t* = 1.0297, df = 275.88, *p* = 0.304) (Figure 1b). Intercept‐only glmm models and average frequencies highlight that shifts between 1950 and 2000 were driven by an overall increase in *Acer negundo* L., *Acer saccharum, Acer rubrum* L., *Populus tremuloides* Michx., *Prunus serotina* Ehrh., and *Celtis occidentalis* L. Declines were primarily in *Quercus rubra* L., *Quercus velutina* Lam., and *Quercus alba* L. (Figure 2, Table S2). 2000s forests were functionally and phylogenetically dissimilar from their 1950s composition, with mean functional similarity of only 0.735 across sites (Figure 3a,b). SES values for functional (*t* = −1.8498, df = 140, *p =* 0.06646) and phylogenetic (*t =* −9.997, df = 140, *p* < 0.001) similarity also reveal that sites are even less similar than would be expected given the degree of taxonomic dissimilarity (Figure 3c,d). Considering how Bray–Curtis dissimilarity is driven primarily by losses of *Quercus* L. and increases in *Acer* L., *Celtis* L., and *Prunus* L., we can also characterize these species as drivers of shifts in functional and phylogenetic similarity in southern WI forests.

### 3.2 Overstory Species Trait Variation

**FIGURE 4:**
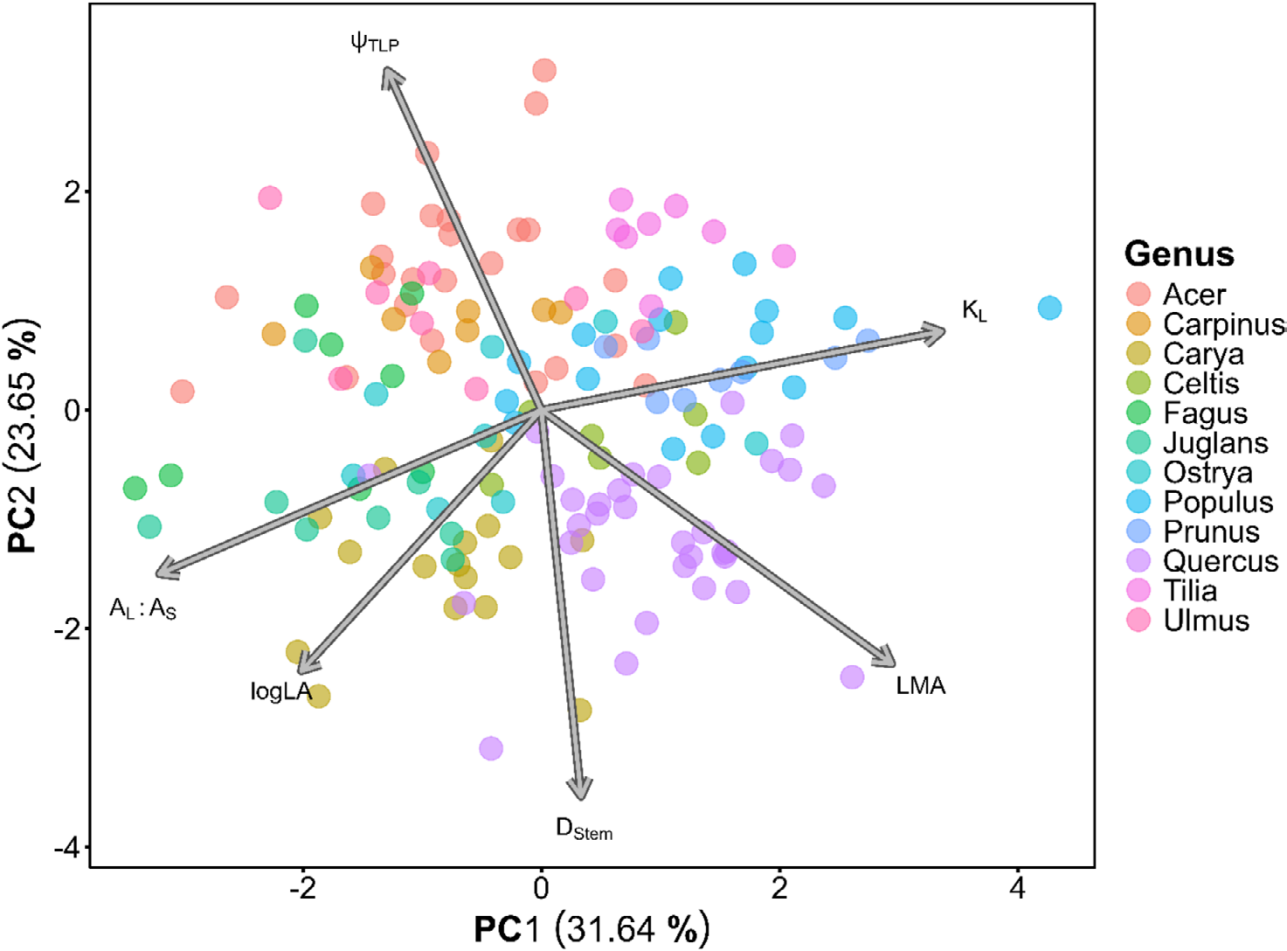
A trait PCA of all 19 species measured, accounting for ~60% of the variance in the trait dataset. D_Sap_, K_S_, and LA were excluded from the PCA due to high degrees of correlation with other traits. Each dot represents an individual, with color denoting genus. Increasing (less negative) Ψ_TLP_ values denote lower drought tolerance.

**FIGURE 5:**
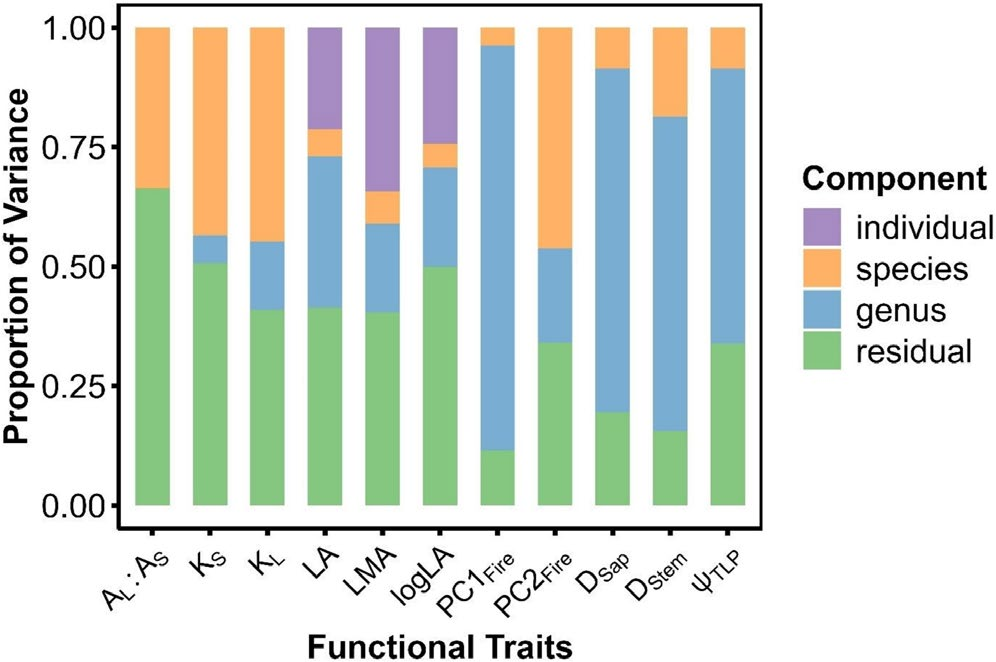
Variance decomposition for each major trait of interest derived from glmm using a Gamma distribution with a log link function. Only LA, LMA, and logLA have individual as a random effect due to intraindividual sampling efforts.

Species trait syndromes varied substantially (Figure S4). The two major trait PCA axes explain roughly 60% of the variation among our species, being driven largely by LMA, Ψ_TLP_, LA, and A_L_A_S_ (Figure 4). Of the traits assessed, A_L_A_S_, K_S_, and K_L_ showed no phylogenetic signal, while bark thickness demonstrated only weak phylogenetic signal. Pagel's *λ* highlighted phylogenetic signal in LMA, LA, D_sap_, D_stem_, Ψ_TLP_, PC1_fire_ and bark thickness (*λ* > 0.5) (Table S3). Variance decomposition analyses corroborate these findings, with the addition of highlighting a high degree of interspecific variation in A_L_A_S_ (Figure 5). All other traits demonstrate a large amount of intergeneric variation, with minor components of variance explained at the species and individual level. A_L_A_S_, K_S_, and K_L_ all demonstrate high levels of residual variation between measurements, and intra‐individual trait variation accounted for ~25% of total variation for LA, logLA, and LMA (Figure 5). The flammability PCA resulted in two axes that explained 55.2% and 17.7% of the variation in burn data (S2). Much of the variation in PC1_fire_, which correlated with flame height, intensity, and duration, occurred at the genus level as opposed to interspecific variation. PC2_fire_, correlated with smoldering and consumption behavior, showed more variation at the species level (Figure 5).

### 3.3 Trait Modeling and CWM Analyses

**FIGURE 6:**
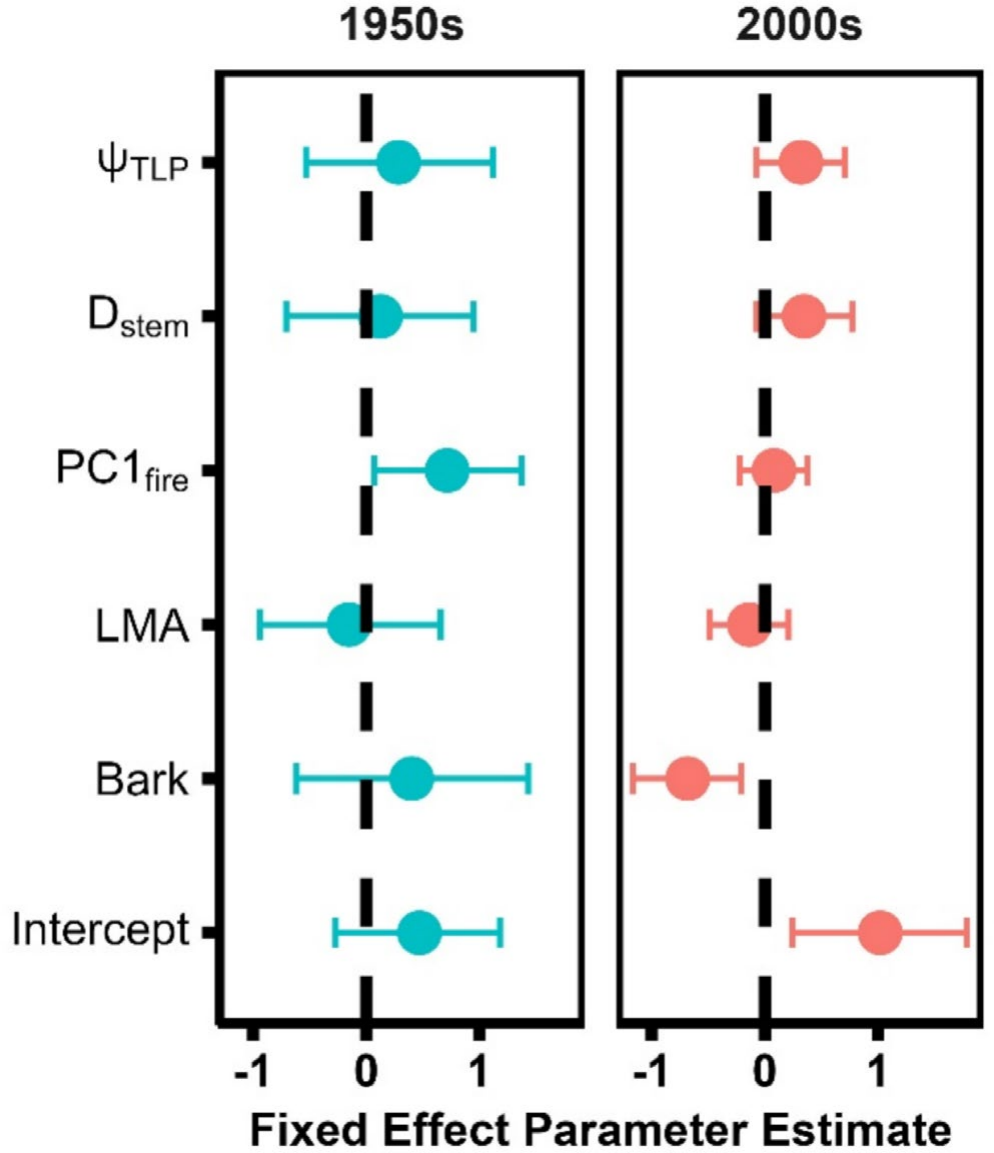
Posterior distributions for trait fixed effect parameters for both 1950s (left) and 2000s (right), with 95% confidence intervals shown.

**FIGURE 7:**
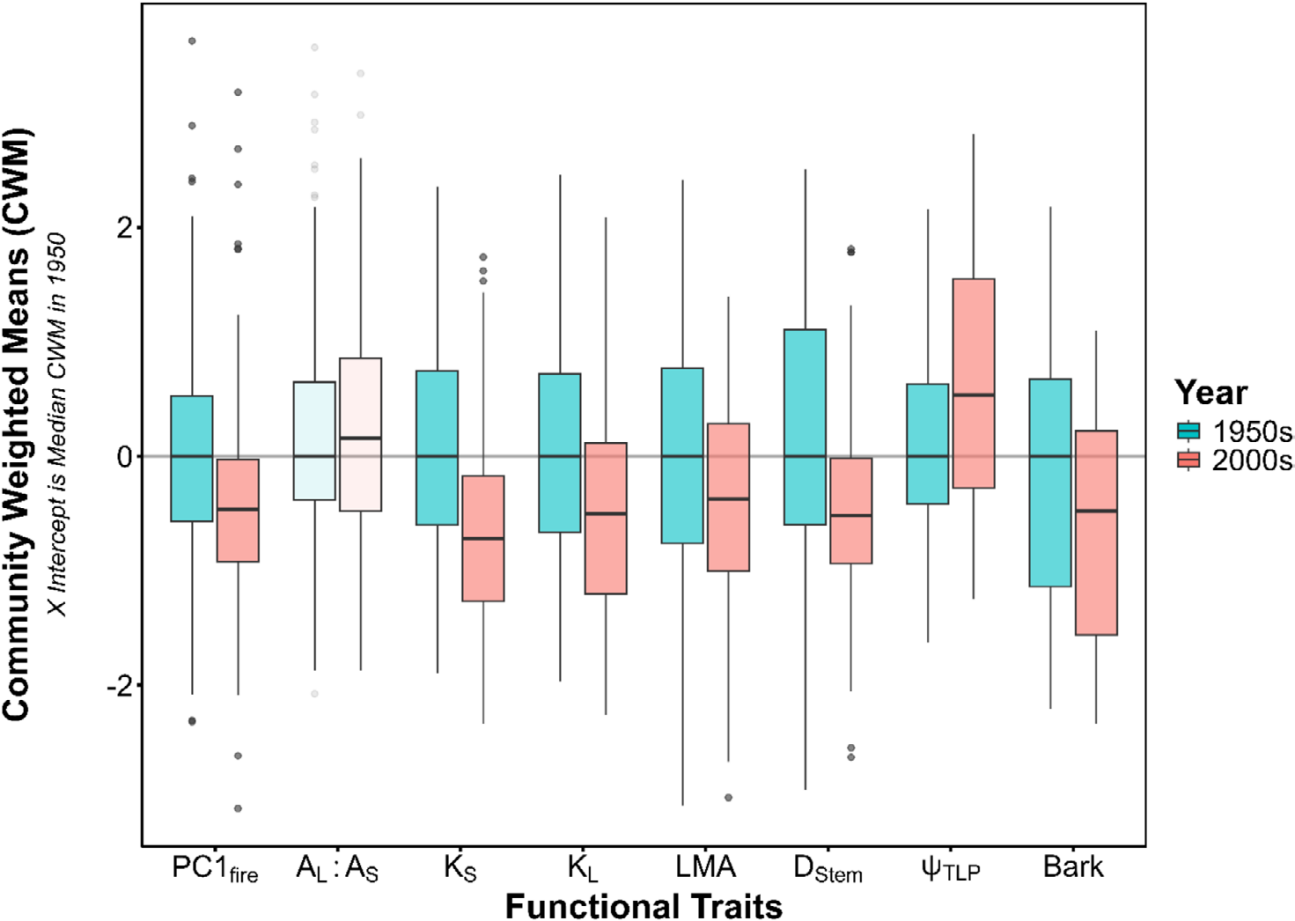
CWM values for all traits at all 138 sites in both 1950s (blue) and 2000s (red). Significance values are derived from *t*‐tests of median CWM values compared to a distribution of values from 1000 random permutations of CWM values within surveying efforts, with opaque boxplots denoting significantly changing CWM over time.

Significant phylogenetic clustering was detected in both the 1950s and 2000s surveys (Tables 2, S3). When traits were incorporated into the models, their inclusion reduced 40.8% of the variance explained by phylogenetic clustering in the 1950s and 19.6% of the variance explained by clustering in the 2000s (Table 2). In the 1950s, PC1_fire_ had a slight positive effect on frequency, while bark thickness had a negative effect on frequency in the 2000's (Figure 6). After randomized permutation tests of site pairwise *t*‐tests, significant negative shifts in CWM for K_S_, K_L_, PC1_Fire_, LMA, D_Sap_, D_Stem_, and bark thickness were identified between the 1950s and 2000s, while Ψ_TLP_ significantly increased (i.e., became less negative) across the landscape over time. CWM values of A_L_A_S_ and logLA did not significantly shift between 1950s and 2000s (Figure 7).

**TABLE 2:** Random effect posterior distributions for (a) 1950s models and (b) 2000s models.

	With traits	Without trait|site random effects	% reduction
**(a) 1950s dataset**			
1|Species	2.029	1.708	
1|Species__	< 0.001	< 0.001	
1|Species__@Site	0.368	0.622	40.83%
LMA|Site	0.363		
Bark|Site	0.189		
TLP|Site	< 0.001		
Stem|Site	< 0.001		
PC1fire|Site	0.058		
1|Site	< 0.001	< 0.001	
Zero inflation term	0.124	0.111	
**(b) 2000s Dataset**			
1|Species	< 0.001	< 0.001	
1|Species__	0.286	0.270	
1|Species__@Site	0.494	0.615	19.64%
LMA|Site	0.164		
Bark|Site	0.138		
TLP|Site	0.036		
Stem|Site	< 0.001		
PC1fire|Site	< 0.001		
1|Site	< 0.001	< 0.001	
Zero inflation term	0.061	0.068	

*Note:* Zero‐inflation terms and reductions in phylogenetic signal are highlighted in green. Traits included in models are those that demonstrate phylogenetic signal and reduced model WAIC when added individually (Table S5). My data repository is officially publically available at https://doi.org/10.5061/dryad.j3tx95xrx.

## 4 Discussion

### 4.1 Dissimilarity and Species Trends

The previous work of Rogers et al. (2008) highlighted the changing composition of canopies in southern Wisconsin between 1950s and 2000s and proposed the general trend of forest mesophication for Wisconsin forests (Hanberry 2019). Our results expand on their conclusions and extend such arguments of mesophication to include significant phylogenetic and functional patterns. Within this species cohort, significant increases in *Acer* and declines in *Quercus*, particularly *Q. alba* and *Q. velutina*, primarily drove both taxonomic and functional dissimilarity (Figures 1, 2, 3, Table S2). Curiously, *Q. macrocarpa* frequency remained unchanged between the 1950s and 2000s, despite being characterized as a species well adapted to fire‐prone savanna systems (Figure 2) (Leach and Givnish 1999; Yantes et al. 2023). The largest increases in species frequency were seen in *C. occidentalis* and *A. negundo* (Figure 2, Table S2). Both of these species are notable for being particularly rare in upland forests in the 1950s (Curtis 1959). *P. serotina* , *P. tremuloides* , *A. rubrum* , and *C. cordiformis* also contributed to southern forest temporal dissimilarity (Figure 2, Table S2). *P. serotina* and *P. tremuloides* have long been identified as early successional, quick growing species capable of reaching the canopy when fire pressure is reduced (Reich et al. 1990b). Slower growing species like *C. caroliniana* , and *C. cordiformis* highlight that such shifts in canopy structure are not just successional results of fire suppression but potentially extend to the creation of altered understory conditions that facilitate the recruitment of late‐successional, fire intolerant species (Peet and Loucks 1977; Walters and Reich 1996). *C. caroliniana* , and *C. cordiformis* only being present in 2000s canopy surveys corroborate this perspective of ‘effect’ functional traits of canopy species substantially altering understory growing conditions (Lavorel and Garnier 2002) (Figure 2).

### 4.2 Trait Syndromes and Variation

With much of the variance in measured traits occurring at the genus level, major genera (*Acer* and *Quercus*) showed separation in terms of LMA, Ψ_TLP_, bark thickness, and D_Stem_ in their trait syndromes (Figure 4, Figure S4). We also observed evidence of large amounts of intraspecific, intraindividual, and residual trait variation, especially for A_L_A_S_ and hydraulic conductivity measurements (Figure 5). As a result, caution should be exercised in using such traits to characterize species acquisition strategies in temperate deciduous trees (S. M. Gleason et al. 2016, Figures 4, 5). While our efforts demonstrate the explanatory value in using species‐mean trait values to identify functional trends in temperate deciduous forests, these results also potentially mask the importance of intraspecific trait variation (Givnish and Montgomery 2014; Westerband et al. 2021). Trait data were collected from open‐grown individuals within a limited geographic range, potentially reducing the extent of intraspecific trait variation captured by these trait analyses (Funk et al. 2017; Westerband et al. 2021). Some species, such as *A. negundo* and *A. rubrum* are reported to exhibit a higher degree of trait plasticity that, when coupled with high allocation to early reproduction, facilitates establishment in historically fire‐prone habitats (Abrams 1998). Future work devoted to quantifying variation at multiple taxonomic scales would more explicitly identify how species compete and grow in differing environmental conditions, providing better insight into the adaptive significance of trait syndromes and how trait plasticity and acclimation may shape our changing Wisconsin forests (L. D. Anderegg et al. 2018; Di Biase and Fattorini 2025).

### 4.3 Phylogenetic Modeling

Phylogenetic models from both the 1950s and 2000s demonstrate a significant amount of variation in species composition explained by phylogenetic clustering (Tables 2, S1). These results indicate that though species composition has significantly changed over time, closely related species still tend to occupy similar sites (Figures 1, 5; Table S1). Since trait fixed effects were mostly insignificant, the clustering seen in the 2000s reflects the phylogenetic clustering of the 1950s as opposed to novel patterns of phylogenetic attraction resulting from mesophication (Table 2, Figure 6). When traits were incorporated as random effects, they accounted for less variance in 2000s models (Table 2), indicating that the clustering seen in 2000s is due to phylogenetic adaptations not accounted for by the traits modeled. Unmeasured species‐specific adaptations to shade, for example, likely contribute to these patterns of phylogenetic clustering, with seedling physiology and light‐capture strategies tied to allometry and resource acquisition being of central importance (Craine and Reich 2005; Kaelke et al. 2001; Kobe et al. 1995; Pacala et al. 1994). *A. negundo* and *A. rubrum* are increasing dramatically in upland forests, while the capacity of traits in explaining phylogenetic clustering is diminishing (Table 2, Figure 2). Considering how forests are becoming functionally dissimilar over time, more traits associated with shade tolerance, reproduction, and germination in low‐light environments may better explain contemporary phylogenetic clustering (Table 2, Figure 3).

In 1950s models, PC1_fire_ had a positive impact on tree species frequency throughout southern Wisconsin forests, highlighting the historical prevalence of species that produce highly flammable litter (Figure 6) (Bray and Curtis 1957). In the 2000s, the negative modeled relationships between bark thickness and frequency and insignificant impact of PC1_fire_ identify the diminished representation of fire‐adapted trees throughout regional forests (Table 2). Bark thickness, especially within *Quercus‐*dominated fire systems like eastern US forests, serves as a key trait in understanding fire tolerance (J. F. Jackson et al. 1999; Pausas 2015). Our results also highlight the increased prevalence of thin‐barked species, including members of *Acer*, *C. occidentalis* , *Ulmus rubra* Muhl, and *P. serotina* (Tables S2, S4). Thinner bark can increase the risk of thermal damage to the vascular cambium, though such damage may only lead to top‐killed individuals (Michaletz and Johnson 2007). Temperate tree species exhibit many adaptations to fire, with resprouting as a common trait among many of the species increasing in Wisconsin forests, including *A. negundo, A. rubrum, P. serotina*, and *Tilia americana* L. (P. J. Clarke et al. 2013; Kruger and Reich 1993). Such fire‐adaptations highlight that meeting historical benchmarks of community composition requires more than simple reintroduction of fire as an ecological disturbance, but an understanding of the physiological and functional responses of tree species to regular fire and conscious application of species removal strategies into management.

Interestingly, Ψ_TLP_ and LMA show no overall effect on species frequency in either the 1950s or 2000s, meaning both surveying efforts reveal broad representation across each trait. Turgor loss point provides insight into the drought tolerance of species, but does little to inform our understanding of drought *avoidance*. Temperate deciduous tree species with higher Ψ_TLP_ tend to avoid drought by closing stomata and eliminating transpiration costs at higher water potentials than species with more negative Ψ_TLP_ (Bartlett et al. 2014; Sala et al. 2012; Zhu et al. 2018). While Ψ_TLP_ informs the relationships between carbon fixation, transpiration, and drought, the ability of species to persist and survive drought is more complex, involving other aspects of survival, including root physiology, non‐structural carbohydrates, and allometry (Bond‐Lamberty et al. 2002; Hoffmann et al. 2011; McCulloh et al. 2019). Furthermore, recent advances in species‐level and seasonal variation in Ψ_TLP_ show the ability of species to shift their Ψ_TLP_ throughout the growing season, drought events, and lifetime (Eisley and Wolfe 2024). Even so, Ψ_TLP_ still remains an effective and insightful metric for gauging species‐level responses to dry conditions, and our results provide support for proposed shifts in drought tolerance as a facet of forest mesophication (W. R. Anderegg et al. 2016; Maréchaux et al. 2015; Meinzer et al. 2016). LMA has been negatively correlated with other aspects of acquisitive leaf habit, and widely implicated as a major source of trait variation between mesophytes and pyrophytes via proposed adaptations to shade and drought (Givnish 1988; Hanberry 2019). Significant LRTs and WAIC of models including LMA|site and Bark|site random effects greatly improve model fit, indicating that species with differing LMA and bark thickness vary in frequency at different sites. Given their phylogenetic signal, these traits are likely responsible for the reduction in variance explained by phylogenetic clustering of species at particular sites (Tables 2, S5). With bark thickness being informative for fire tolerance and LMA often being considered indicative of a more acquisitive growth strategy, this historic and contemporary clustering likely reflects historic disturbance regimes and environmental conditions at each site, known to vary considerably throughout southern Wisconsin (Clayton and Moran 1982; Curtis 1959; Shea et al. 2014). Further modeling efforts that incorporate environmental variables may identify strong trait‐environment effects on frequency (Table 2). While forests are shifting in functional similarity and composition, patterns related to acquisitive trait syndromes are shown to be shaped by site‐level conditions and not yet reflected in overall species frequency across the region or in species‐level modeling (Table 2, Figure 6). Overall, these modeling results highlight the persistence of phylogenetic clustering in WI forests, broad declines in thick‐barked and flammable species, and the diminished explanatory value of traits in 2000s clustering patterns (Figure 6). While broadly supporting mesophication in terms of fire tolerance, these patterns clarify lines of inquiry into the functional importance of low‐light adaptation. Specifically, the low explanatory power of traits in explaining 2000s clustering highlights the need for a more comprehensive and mechanistic understanding of shade tolerance variation in temperate broadleaf species, traits already known to impact seedling growth and survival in temperate forest understories (Craine and Reich 2005).

### 4.4 Regional and Global Implications

Our results show significant shifts toward communities with lower LMA, higher Ψ_TLP_, less flammable litter, and decreased bark thickness that broadly support the mesophication hypothesis (Figure 7). When considered with our modeling techniques and analyses of functional similarity, these results suggest our forests are moving toward a less fire‐tolerant, more drought‐avoidant trait syndrome, though we are likely documenting a lagging response to fire suppression, with shifts not yet representative in larger regional models of species frequency (Table 2, Figures 6, 7). Notably, foliar data highlights an important consideration for ongoing forest management and restoration within the prairie‐woodland ecotone of the Midwest (Bohman et al. 2021). While the importance of liberating oak savannas from mesic forest species is well understood, forests with increasingly thin leaves and thin‐barked trees highlight a potential difficulty in propagating and promoting fire in forest understories. Particularly, the reintroduction of fire is integral to fostering the high plant diversity historically seen in oak savanna and oak woodland communities (Leach and Givnish 1999; Yantes et al. 2023). Likewise, thinner leaves with low LMA can promote an acceleration of nutrient cycling into the soil, further altering soil nutrient concentration, texture, and moisture holding capacity (Rosenfield et al. 2020). Beyond removing mesic forest species, planting, and promoting the growth of oaks can foster future fire and nutrient‐cycling dynamics more akin to historic oak savannas and dry woodlands. In more mesic forests, careful management for mesic species with proposed adaptability will also maintain ecosystem services and promote mesic understory persistence (Iverson et al. 2019; Peters et al. 2020). These functional findings also reinforce questions related to proposed feedback loops inherent to the succession described by mesophication and future forest management and conservation (Nowacki and Abrams 2008).

Considering predictions of higher temperatures, infrequent and intense precipitation across mid‐latitudes, and elevated potential for drought in subsequent decades, the outlined functional trajectory of regional forests provides additional context for future forest health and resiliency more broadly (Hoffmann et al. 2011; Swanston et al. 2018; Wisconsin Initiative on Climate Change Impacts 2011). While climate adaptation for temperate forests is at the forefront of many national and international organizations, most acknowledge that we have key gaps in our understanding of how forests have already functionally changed (Allen et al. 2015; Iverson et al. 2019; Matthews et al. 2011; Peters et al. 2020; B. M. Rogers et al. 2017). While international efforts to bolster forest resiliency continue to incorporate species life history into vulnerability assessments (Aubin et al. 2018; Bohman et al. 2021; Brandt et al. 2014; Reidmiller et al. 2018; Sousa‐Silva et al. 2018; Swanston et al. 2018), our approach quantifies and connects dissimilarity with functional traits that are informative and applicable to temperate forests globally, both from an assessment and adaptive management perspective.

## 5 Conclusion

Driven by increasing *Acer* and declining *Quercus*, upland forest communities in WI have made notable functional and phylogenetic shifts in the latter half of the 20th century. Despite this, functional dissimilarity is accompanied by statistical support for persistent phylogenetic clustering of species from the 1950s to 2000s and the overall decreasing frequency of fire‐adapted species. Dominant overstory species trait syndromes demonstrate strong phylogenetic signal at the genus level, separating primarily in terms of LMA, Ψ_TLP_, D_stem_, litter flammability, and bark thickness. Though able to explain phylogenetic clustering in both survey efforts, traits explain less of the phylogenetic clustering in the 2000s, implying the need to measure additional traits, specifically those related to low‐light assimilation. Across the landscape, sites have significantly shifted toward a more drought‐avoidant, fire‐intolerant composition, highlighting potential shifts in fire survival, fire facilitation, and nutrient dynamics in Wisconsin woodlands. These analyses quantify various proposed functional impacts of mesophication throughout southern Wisconsin. At the ecosystem level, our findings highlight an altered fire regime in temperate woodland systems and provide a sound, regional basis for analyses of the alteration of nutrient cycling and other ecosystem services. This additional context for mesophication improves woodland management and strengthens the call for ongoing monitoring to determine the velocity and trajectory of these functional trends in temperate woodlands. Though temperate deciduous forests around the globe will experience disparate impacts of climate change and development, the functional and phylogenetic trends identified in testing the mesophication hypothesis demonstrate the flexibility and value of a mixed‐modeling approach to analyzing changing temperate forests.

## Author Contributions

**Sam W. Anderson:** conceptualization (lead), data curation (lead), formal analysis (lead), investigation (equal), methodology (equal), project administration (lead), visualization (lead), writing – original draft (lead), writing – review and editing (equal). **David A. Rogers:** conceptualization (supporting), data curation (supporting), formal analysis (supporting), investigation (supporting), methodology (supporting), resources (supporting), validation (supporting), writing – review and editing (supporting). **Katherine A. McCulloh:** conceptualization (equal), investigation (supporting), methodology (supporting), project administration (supporting), resources (supporting), supervision (supporting), validation (supporting), visualization (supporting), writing – review and editing (supporting).

## Funding

This work was supported by the National Science Foundation Graduate Research Fellowship Program, 2137424.

## Conflicts of Interest

The authors declare no conflicts of interest.

## Supporting information


**Data S1:** ece373323‐sup‐0001‐Supinfo.docx.
**Figure S1:** A map showing all study site locations (orange dots) that provided community composition data used in these analyses. The state of Wisconsin is highlighted in yellow. The upper right map shows the continental context for the Great Lakes region in North America, while the lower left map provides a closer look at the Great Lakes region and the distribution of study sites.
**Figure S2:** Flammability Trait PCA for overstory species, with color representing species. PC1 can be interpreted as fire intensity, with higher PC1 values denoting more flammable species. PC2 corresponds to fire persistence, correlated with the duration of smoldering and leaf curling.
**Figure S3:** Histograms showing pairwise shifts in site‐level species richness (left) and tree diversity (Shannon's entropy *q* = 1). Histograms also include data on northern forest sites that were not analyzed in the rest of this publication.
**Figure S4:** PCA of Species mean trait data, incorporating PC1_Fire_ and Bark thickness (mm) data into the trait data summarized in Figure 5.
**Table S1:** Table of pglmm model results testing model improvement when including phylogenetic attraction terms. Models included only random effects shown.
**Table S2:** Table of SIMPER analysis values comparing sites in the 1950s and 2000s (from the r package *vegan* 2.6–8). Species that contribute significantly to the dissimilarity of communities between the 1950s and 2000s are highlighted. Columns denote the average contribution of this species to the average dissimilarity between observations from the 1950's and 2000's (average), the standard deviation of the contribution of this species to dissimilarity (sd), the ratio of average to sd, akin to a coefficient of variation (ratio), the average abundance of this species in each of the two groups, group A being the 1950's surveys (ava) and group B (avb) being the 2000's surveys, and a permutation‐based *p*‐value, showing the probability of getting a larger or equal average contribution for each species if the grouping factor was randomly permuted (*p*).
**Table S3:** Results of phylogenetic signal using species trait means in the R package *phytools*. Models Testing for Significance of Phylogenetic Attraction. Highlighted traits show a Pagel's *λ* > 0.5.
**Table S4:** Table showing fixed effect posterior distributions and the 95% confidence intervals displayed in Figure 6. Highlighted parameter estimates do not encompass 0.
**Table S5:** WAIC from pglmm models excluding (Trait|Site) random effects for all traits that demonstrate phylogenetic signal Table S2, used to justify trait models presented in Table 2.

## Data Availability

Functional trait, litter flammability, and community composition data can be found at https://datadryad.org/dataset/doi:10.5061/dryad.j3tx95xrx. Phylogeny of Wisconsin angiosperms can be found at https://datadryad.org/stash/dataset/doi:10.5061/dryad.kf6q10b (Spalink et al. 2018).
