# Supplementary material for: Functional and Phylogenetic Implications of Canopy Mesophication in Temperate Hardwood Forests
Source: Ecol Evol. 2026 Apr 2;16(4):e73323. doi: 10.1002/ece3.73323 (PMC13045328; doi:10.1002/ece3.73323)
Supplement: Supplementary file 1 — Data S1: ece373323‐sup‐0001‐Supinfo.docx. Figure S1: A map showing all study site locations (orange dots) that provided community composition data used in these analyses. The state of Wisconsin is highlighted in yellow. The upper right map shows the continental context for the Great Lakes region in North America, while the lower left map provides a closer look at the Great Lakes region and the distribution of study sites. Figure S2: Flammability Trait PCA for overstory species, with color representing species. PC1 can be interpreted as fire intensity, with higher PC1 values denoting more flammable species. PC2 corresponds to fire persistence, correlated with the duration of smoldering and leaf curling. Figure S3: Histograms showing pairwise shifts in site‐level species richness (left) and tree diversity (Shannon's entropy q = 1). Histograms also include data on northern forest sites that were not analyzed in the rest of this publication. Figure S4: PCA of Species mean trait data, incorporating PC1Fire and Bark thickness (mm) data into the trait data summarized in Figure 5. Table S1: Table of pglmm model results testing model improvement when including phylogenetic attraction terms. Models included only random effects shown. Table S2: Table of SIMPER analysis values comparing sites in the 1950s and 2000s (from the r package vegan 2.6–8). Species that contribute significantly to the dissimilarity of communities between the 1950s and 2000s are highlighted. Columns denote the average contribution of this species to the average dissimilarity between observations from the 1950's and 2000's (average), the standard deviation of the contribution of this species to dissimilarity (sd), the ratio of average to sd, akin to a coefficient of variation (ratio), the average abundance of this species in each of the two groups, group A being the 1950's surveys (ava) and group B (avb) being the 2000's surveys, and a permutation‐based p‐value, showing the probability of getting a larger [file ECE3-16-e73323-s001.docx]

Forest Mesophication Supplemental


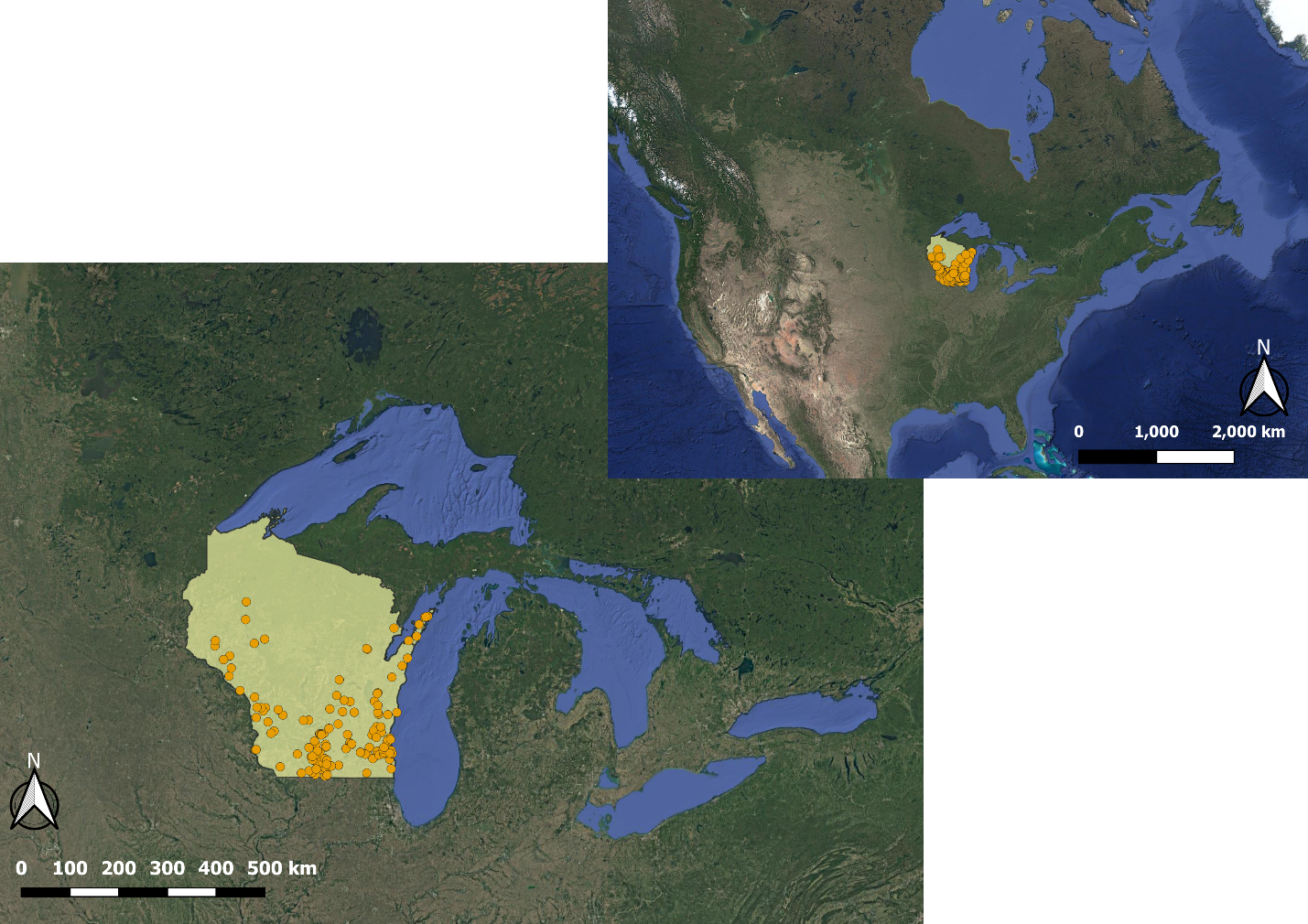


S1: A map showing all study site locations (orange dots) that provided community composition data used in these analyses. The state of Wisconsin is highlighted in yellow. Upper right map shows continental context for the Great Lakes region in North America, while lower left map provides a closer look at the Great Lakes region and distribution of study sites.


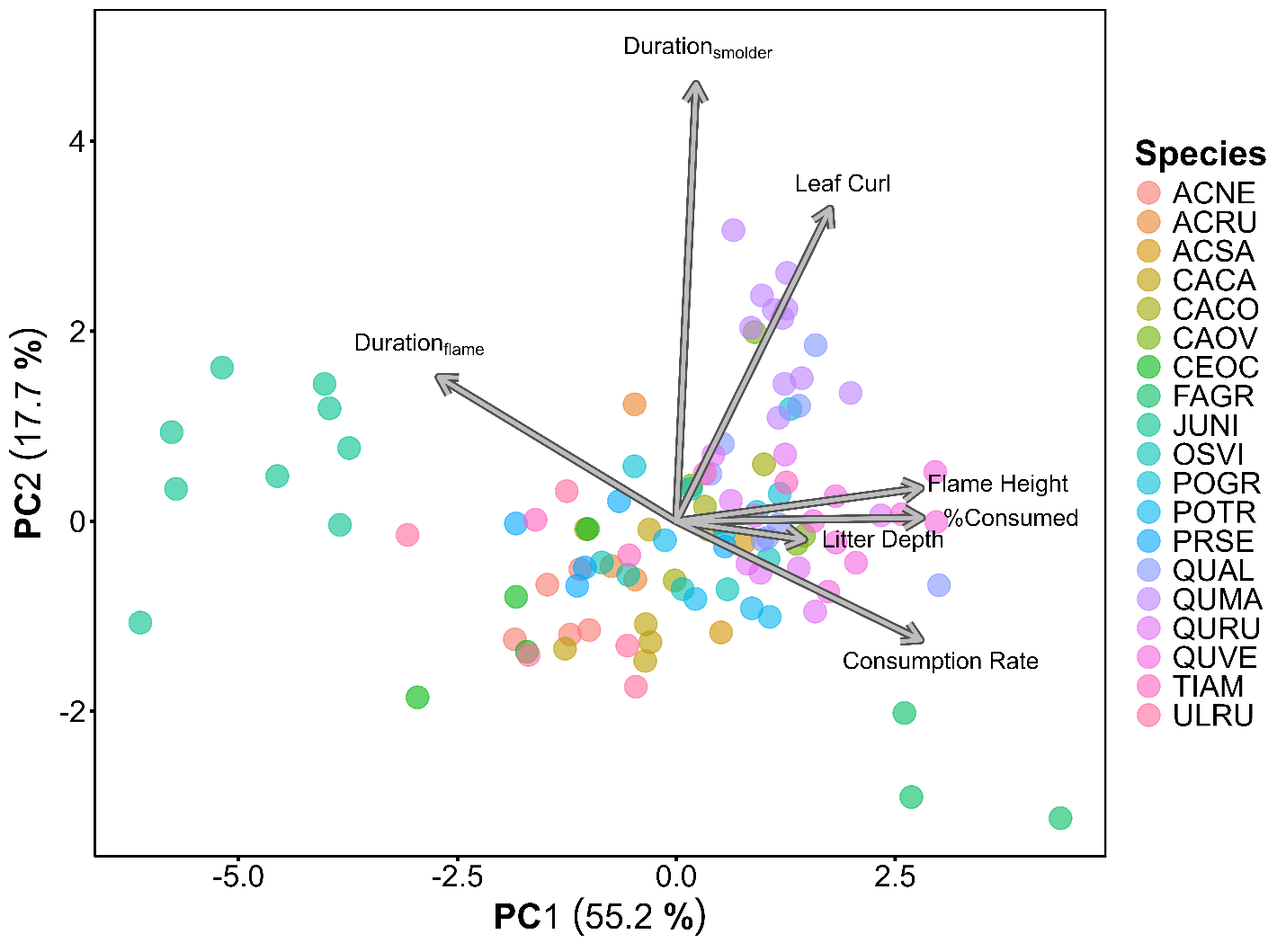


S2: Flammability Trait PCA for overstory species, with color representing species. PC1 can be interpreted at fire intensity, with higher PC1 values denoting more flammable species. PC2 corresponds to fire persistence, correlated with the duration of smoldering and leaf curling.

Table S1: Table of pglmm model results testing model improvement when including phylogenetic attraction terms. Models included only random effects shown

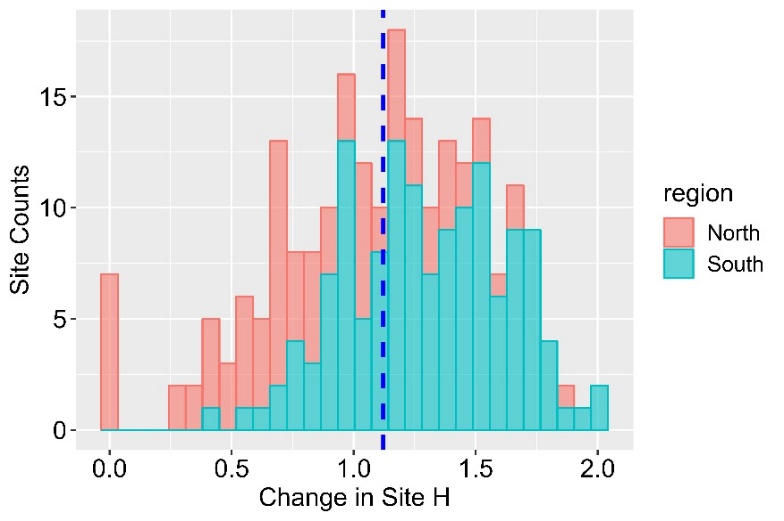

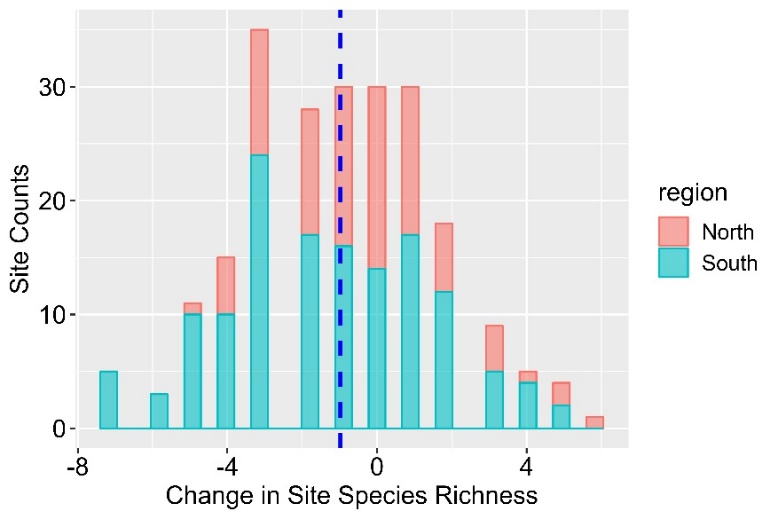


S3: Histograms showing pairwise shifts in site-level species richness (left) and tree diversity (Shannon’s entropy q=1). Histograms also include data on northern forest sites that were not analyzed in the rest of this publication.

Table S2: Table of SIMPER analysis values comparing sites in the 1950s and 2000s (from r package *vegan* 2.6-8). Species that contribute significantly to the dissimilarity of communities between the 1950s and 2000s are highlighted. Columns denote the average contribution of this species to the average dissimilarity between observations from the 1950’s and 2000’s (average), the standard deviation of the contribution of this species to dissimilarity (sd), the ratio of average to sd, akin to a coefficient of variation (ratio), the average abundance of this species in each of the two groups, group A being the 1950’s surveys (ava) and group B (avb) being the 2000’s surveys, and a permutation-based *p*-value, showing the probability of getting a larger or equal average contribution for each species if the grouping factor was randomly permuted (p)

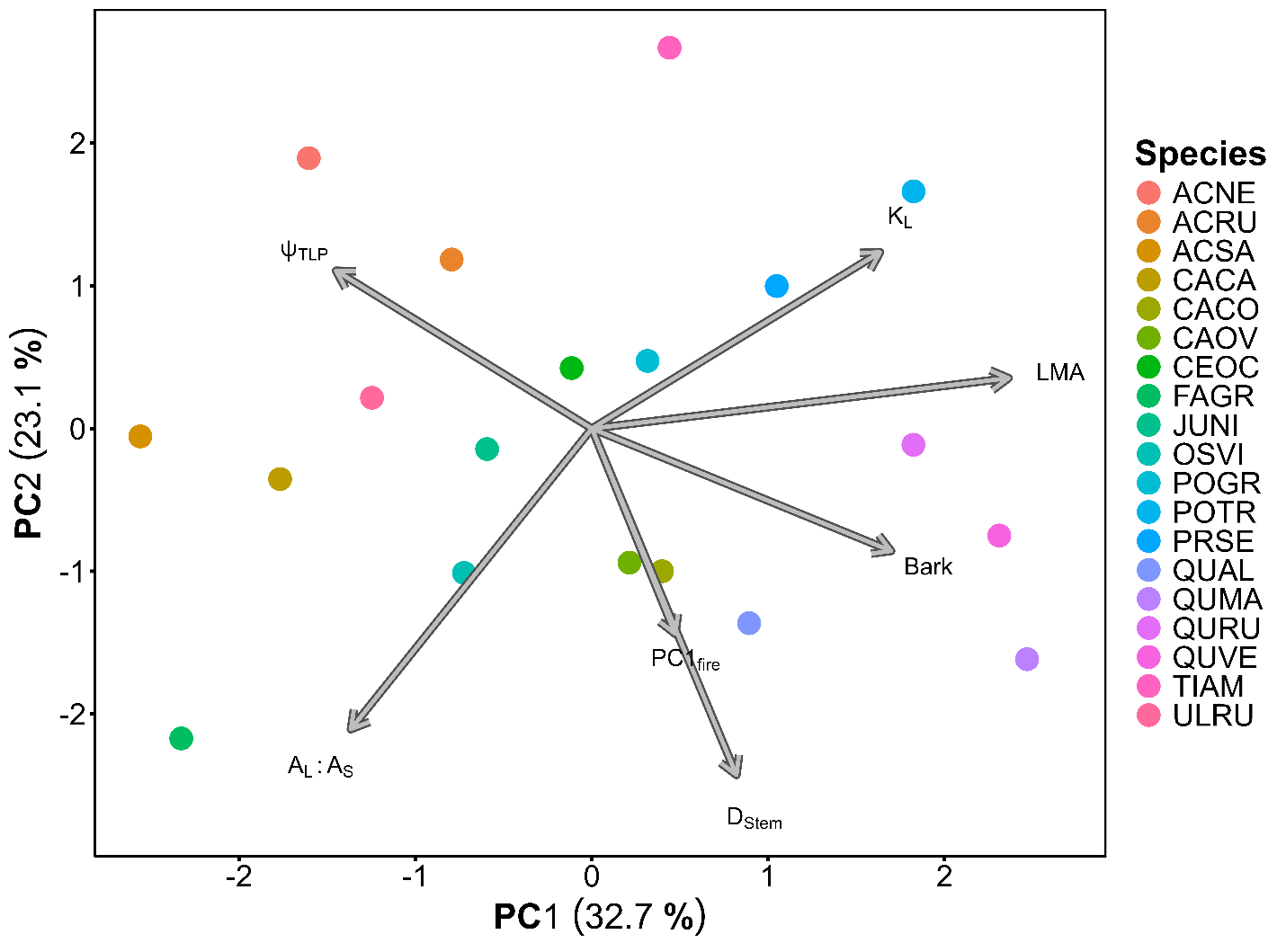


S4: PCA of Species mean trait data, incorporating PC1_Fire_ and Bark thickness (mm) data into trait data summarized in Fig.5

Table S3: Results of phylogenetic signal using species trait means in R packages *phytools.*

Models Testing for Significance of Phylogenetic Attraction. Highlighted traits show a Pagel’s λ > .5

Table S4: Table showing fixed effect posterior distributions and the 95% confidence intervals displayed in Figure 6. Highlighted parameter estimates do not encompass 0.

Table S5: WAIC from pglmm models excluding (Trait|Site) random effects for all traits that demonstrate phylogenetic signal Table S2, used to justify trait models presented in Table 2.

| **Trait** | **1950s** | **2000s** |
| --- | --- | --- |
| **Intercept-only** | 12089.55 | 12902.71 |
| **LMA** | 9993.153 | 11472.37 |
| **Bark** | 10609.593 | 11168.71 |
| **Dstem** | 10797.4 | 12040.52 |
| **TLP** | 11212.82 | 11334.21 |
| **PC1** | 11018.91 | 12035.9 |
